# Platform Effects on Public Health Communication: A Comparative and National Study of Message Design and Audience Engagement Across Twitter and Facebook

**DOI:** 10.2196/40198

**Published:** 2022-12-20

**Authors:** Nic DePaula, Loni Hagen, Stiven Roytman, Dana Alnahass

**Affiliations:** 1 School of Information Sciences Wayne State University Detroit, MI United States; 2 School of Information University of South Florida Tampa, FL United States; 3 Department of Radiology University of Michigan Ann Arbor, MI United States; 4 School of Medicine Wayne State University Detroit, MI United States

**Keywords:** platform effects, COVID-19, social media, health communication, message design, risk communication, Twitter, Facebook, user engagement, e-government

## Abstract

**Background:**

Public health agencies widely adopt social media for health and risk communication. Moreover, different platforms have different affordances, which may impact the quality and nature of the messaging and how the public engages with the content. However, these platform effects are not often compared in studies of health and risk communication and not previously for the COVID-19 pandemic.

**Objective:**

This study measures the potential media effects of Twitter and Facebook on public health message design and engagement by comparing message elements and audience engagement in COVID-19–related posts by local, state, and federal public health agencies in the United States during the pandemic, to advance theories of public health messaging on social media and provide recommendations for tailored social media communication strategies.

**Methods:**

We retrieved all COVID-19–related posts from major US federal agencies related to health and infectious disease, all major state public health agencies, and selected local public health departments on Twitter and Facebook. A total of 100,785 posts related to COVID-19, from 179 different accounts of 96 agencies, were retrieved for the entire year of 2020. We adopted a framework of social media message elements to analyze the posts across Facebook and Twitter. For manual content analysis, we subsampled 1677 posts. We calculated the prevalence of various message elements across the platforms and assessed the statistical significance of differences. We also calculated and assessed the association between message elements with normalized measures of shares and likes for both Facebook and Twitter.

**Results:**

Distributions of message elements were largely similar across both sites. However, political figures (*P*<.001), experts (*P*=.01), and nonpolitical personalities (*P*=.01) were significantly more present on Facebook posts compared to Twitter. Infographics (*P*<.001), surveillance information (*P*<.001), and certain multimedia elements (eg, hyperlinks, *P*<.001) were more prevalent on Twitter. In general, Facebook posts received more (normalized) likes (0.19%) and (normalized) shares (0.22%) compared to Twitter likes (0.08%) and shares (0.05%). Elements with greater engagement on Facebook included expressives and collectives, whereas posts related to policy were more engaged with on Twitter. Science information (eg, scientific explanations) comprised 8.5% (73/851) of Facebook and 9.4% (78/826) of Twitter posts. Correctives of misinformation only appeared in 1.2% (11/851) of Facebook and 1.4% (12/826) of Twitter posts.

**Conclusions:**

In general, we find a data and policy orientation for Twitter messages and users and a local and personal orientation for Facebook, although also many similarities across platforms. Message elements that impact engagement are similar across platforms but with some notable distinctions. This study provides novel evidence for differences in COVID-19 public health messaging across social media sites, advancing knowledge of public health communication on social media and recommendations for health and risk communication strategies on these online platforms.

## Introduction

### Background

Social media have become integral tools for public health messaging and online communication of health and risk information worldwide [1-3]. As of 2021, in the United States, 72% of adults and 84% of those aged 18-29 years say they use at least 1 social media site [4,5] and the sites are widely adopted by public health agencies [3,6,7]. On social media, public health messages can be shared by users, widening message reach. The public may also like and comment on agency messages, and agencies may directly reply to public comments. Although there are opportunities for public health messaging on these sites, there are also challenges. These sites have been sources of misinformation, especially concerning the COVID-19 pandemic [8,9, 10] and antivaccination propaganda [11,12]. The targeted marketing of health-harming products, such as e-cigarettes [13], has also been problematic. Nevertheless, given their prevalence, public health agencies need to understand the dynamics of these sites to better promote health behavior.

There is ample research on social media use by public health agencies [2,7,14,15,16]. However, studies are generally conducted on one site or another, either Facebook or Twitter. Although studies in other domains abound exploring the distinct affordances or characteristics of different social media sites [17-20], there are few studies examining user engagement with public health messages [13,21] and no analyses of the actual messages posted by public health agencies across social media platforms. Despite the lack of such comparative studies, it is important to understand the media effects, or at least the differences across sites. Studies often use the term “social media” broadly when they only investigate a single platform. However, the stark differences across some platforms are now well researched [22-24], and there has been an explicit call for addressing social media affordances in health communication research [25]. This study thus makes a novel contribution to the literature by comparing public health messaging and audience engagement across two of the most popular platforms in public health communication.

### Public Health Message Design and Audience Engagement

Research on public health messaging on social media has focused on 2 broad areas: (1) the *content* and *purposes* of messages and (2) *audience* (or *user*) *engagement* with the messages. Analyses of message content have focused on “themes,” such as “closures,” “risk factors,” “case updates,” “reassurance,” and others, in various pandemic and crisis contexts [26,27], including the COVID-19 pandemic [7,28]. Analyses of message purposes have discussed the goals of “to inform,” “call to action” [28], increase “self-efficacy” [29], “fight misinformation” [30], and others. However, there is a lack of formalization of message design elements and little consideration for the more objective textual elements of messages, including relevant content, such as the speaker, audience, and types of images in the messages. To address this shortcoming, in this study, we adopted a framework of *textual and media message design elements* that identify the various objective characteristics of the text—focusing on the content, not on the purpose—which may be useful for multiple health and risk communication scenarios and related research [31].

Audience or user engagement on social media is often formalized in the platform via a *Like* button, a *Share* button, and a *Comment* function, the content or count of which is appended to the message. Facebook also offers other sentiments or reactions to be expressed that are formalized as buttons and counts (ie, *love*, *care*, *ha-ha*, *wow*, *sad*, and *angry*). Although social media reactions to messages may not directly relate to behavioral intent or actual behavior change, analyses of this engagement provide some insight into public interest in and acceptance of the messaging [25] and may therefore help improve message strategies and message design, what others have termed evidence-based science communication [13]. There is a downside to an overreliance on user engagement as the ultimate goal of social media communication, since user engagement is biased toward positive emotional or high arousal content [23,32, 33]. However, these metrics at least provide some evidence of the quality or success of health promotion and information campaigns on these platforms and can be used to increase message reach [13].

### Platform Effects on Health and Risk Messages

Although studies in the social media literature recognize the distinct *affordances*—the functions or *action possibility* [25]—of these technologies, previous studies on COVID-19 lack a study of message elements across the most popular platforms: Facebook and Twitter [13,25]. Although they are similar, Facebook and Twitter share some key differences. On Facebook, connections of people are bidirectional and termed as “friends.” On Twitter, they are unidirectional; individuals may follow others without being followed by them. This makes Twitter a more public and open platform. However, Facebook is a more popular site, with a marketplace, event calendars, and pages that can be unidirectionally followed [34]. On both Facebook and Twitter, individuals may make posts that include text, hyperlinks, and photos or videos, but the text length of a post is restricted on Twitter to 280 characters. They both have a *newsfeed* that presents users with posts of their friends, or those followed, the organization of which is determined by the platform algorithms [35].

In practice, Facebook is more widely adopted than Twitter across all demographic groups [34]. Twitter has been used as a “news media” [36] and is associated with political news [37]. Twitter has been found to be more used for public information [38], whereas Facebook is used for “shared identities” [24] and “social interaction” [39] and is associated with higher levels of privacy concern and bonding social capital [22]. A recent study of user engagement with antismoking messages found that the message theme (ie, health/appearance/addiction, money, or family) has no impact on the click-through rate (CTR) of messages, but Facebook had the highest and lowest CTR levels and on average higher CTRs than the same messages on Twitter [13], showing that users on Facebook generally engage more than users on Twitter. However, messages on Twitter had a higher website CTR than those in any other platform, indicating that Twitter users are more likely to go to and scroll through the website linked to in the messages [13]. The literature thus supports the notion of Facebook as more of a social interaction platform, whereas Twitter is more of a news-oriented platform.

### Research Objectives and Summary

For this study, we aim to assess differences in public health message design elements and audience engagement with the various message elements across Twitter and Facebook regarding COVID-19 during 1 year of the pandemic. We therefore ask the following research questions (RQs):

- RQ1. How do public health message design elements differ across Twitter and Facebook?
- RQ2. How does audience engagement with public health message elements differ across Twitter and Facebook?

In the following sections, we describe the methods of the study, the results, and the discussion in relation to the literature and provide evidence-based policy recommendations for better-targeted health communication strategies.

## Methods

### Data Collection and Sampling

We identified 11 major federal health agencies in the United States associated with infection prevention and control [40], the major public health agency of each of the 50 US states (plus Washington, DC), and the major local public health agency of each of the largest city/county in the 50 states. We then searched for the official account of these agencies on Twitter and Facebook, as well as their own website. Not all of the largest city/county public health agencies of the states had a Facebook or Twitter presence. From the list of agencies identified, we retrieved all COVID-19–related posts generated in 2020. This period enables an analysis of messages from the beginning of the pandemic through several waves. We then searched for any of the following strings anywhere in any of the posts of all identified agencies: *ncov*, *covid*, *corona*, *pandemic*, or *sars-cov*. To retrieve these posts, we used the standard Twitter application programming interface (API) and the Facebook API via Crowdtangle [41]. Note that the terms “post” and “message” are used here interchangeably. Unless otherwise specified, the term “post” refers to *original posts* and not *retweets* (shared posts) or *replies* (comments on other posts).

On Twitter, we identified 11 federal accounts (with a total of COVID-19–related original posts and retweets), 48 state accounts (with a total of 40,716 posts and retweets), and 33 local accounts (with a total of 20,164 posts and retweets) that matched the criteria. On Facebook, we identified 10 federal accounts (with a total of 3592 posts), 49 state accounts (with a total of 34,930 posts), and 38 local accounts (with a total of 14,356 posts) that matched the criteria. On Facebook, it is more difficult to differentiate original posts from shared posts; the figures just reported for Facebook include both. This data set of all COVID-19–related posts from all identified agencies in 2020 was called the *population data set*.

For manual content analysis, we used a stratified random sampling technique where we sampled 900 posts from Twitter and 900 posts from Facebook proportional to the amount of posts made by agency level (ie, local vs state vs federal), the *sample data set*. The rationale for the sampling was based on similar studies and generating a manageable number of posts to manually code. For example, Reuter et al [13] analyzed a total of 1275 antismoking health messages posted across 3 social media platforms, and Slavik et al [15] used 501 tweets for content analysis of Canadian public health agencies’ messages on Twitter. We should note that for Facebook, our sampling strategy only focused on posts that were shorter than 340 characters (which may include relatively long hyperlinks). This was intended to provide a data set more comparable to Twitter posts, which are restricted to 280 characters (where hyperlinks may be shortened). After removing nonrelated posts, reply posts, and shared posts, or posts without any discernible content, our *final sample* *data set* consisted of a total of 1677 (93.2%) posts (826, 49.3%, original Twitter posts and 851, 50.7%, original Facebook posts) that were coded*.* For Twitter, this included 82 (9.9%) federal posts, 482 (58.4%) state posts, and 262 (31.7%) local posts. For Facebook, this included 60 (7.1%) federal posts, 560 (65.8%) state posts, and 231 (27.1%) local posts. Multimedia Appendix 1 presents the sampled accounts.

### Coding Framework

We adapted an existing framework [31] for the analysis of health and risk communication social media message elements. The framework is based on theories of text analysis [31,42, 43] and social media studies in health and crisis communication [7,15,28,29], including image use in risk communication [44]. These are interdisciplinary studies in the health communication, health informatics, and crisis communication literature. The framework focuses on message elements that are more objective compared to the abstract (eg, “open and transparent message” [45]) and metaphorical (eg, “fighting misinformation” [30]) categories used in the literature—or assuming everything is a “frame” or “theme” [26,27]. Message elements in this framework are composed of *textual* and *media elements*. The framework integrates message elements into 8 major dimensions: *speech function*, *topic*, *threat focus*, *type of resource*, *audience*, *speaker*, *rhetorical tactic*, and *media*. Each of these dimensions includes more granular *message features* (or *elements*). Tables 1 and 2 introduce definitions and examples of the textual and media elements, respectively. The framework is not exhaustive and could be reduced or expanded, as needed. It is conceived for relatively short social media posts, since the analysis focuses on the clause or sentence level, and therefore lengthier documents would be largely more complex to analyze. Further details of the framework and the elements are provided in Multimedia Appendix 2.

**Table 1:** Definitions and examples of message elements: textual.

Textual element		Definition	Example
**Speech function**			
	Representative	Clause in declarative form, describing a behavior, state, or event	“#COVID19 can be spread by people who do not have symptoms”
	Directive	A sentence that directs, commands, or mandates an action, especially via an imperative sentence	“Continue to wear masks” OR “Donate blood.”
	Question	A rhetorical question or question prompt	“Are you looking for work? We are hiring!”
	Expressive	Expression of sentiment by the message speaker (eg, sadness, appreciation)	“Thank you, #EMS heroes, for staying strong”
	Request	Request to participate in research, volunteer, or means to reach an agency	“Call us for questions at this number”
**Topic**			
	Protection	Information about what to do to prevent or treat the issue	“Disinfect things you and your family touch frequently”
	Policy	Actions, policies, or programs of officials, government agencies, or related entities	“Multnomah County is almost ready for reopening schools.”
	Surveillance	Statistics or data about prevalence (eg, cases/deaths)	“Yesterday, there were 85 new deaths”
	Science	Describes or explains a cause, mechanism, or symptom of the issue	“there is no evidence that produce can transmit #COVID19”
	Emergent	Event of emergency concern or immediate priority	“Travelers: DON'T book air travel to NY for just a few days”
**Resource type**			
	Interactive	Interactive service, such as question-and-answer (Q&A) with policy makers or watching live	“FDA will host a virtual Town Hall on 3D printed swabs”
	Material	Testing sites, financial assistance, vaccine provision	“Use our map to find locations for vaccination sites.”
	Corrective	Correction of a rumor, misinformation, or pointing to related resources	“A death previously reported in Warren was incorrect, and has been removed.”
**Focus and audience**			
	Group	Refers to a demographic group (eg, adults, Hispanics) or a vulnerable population	“Cancer patients are among those at high risk of serious illness from a COVID19 infection.”
	Secondary	Consequences of or issues directly related to the main issue	“Many are feeling stressed because of #COVID19.”
	Other language	Message or part of message in another language, including sign language	“Números del #COVID19 en California:”
**Speaker**			
	External	Expert or staff from another agency	“The head of the CDC will speak…”
	Political	Mayor, governor, or other political figure	“Watch the Mayor’s updates on…”
	Expert	Expert or staff of the agency	“Our own Dr. Elinore will discuss the crisis”
	Personality	Nonpolitical or nongovernmental personality, including celebrities or community members	“Juan from Blue Eagles football club speaks about COVID19”
**Rhetorical**			
	Collective	Focus on collective terms to characterize an issue or to address it	“We all need to do our part to combat Covid-19”
	Emphasis	Sentence with an explanation point or with all capitalized directive	“WEAR a mask!”
	Positive	Positive framing of agency action	“We’re making progress is getting vaccines”
	Metaphor	Using metaphors to explain the science or prevention of the issue	“The swiss cheese respiratory virus defense”

**Table 2:** Definitions and examples of message elements: media.

Media element	Definition	Example
Hyperlink	A long or short web URL	https://twitter.com/...
Hashtag	Any term preceded by a # symbol	#COVID-19 #WearAMask
Text-in-image	Image with additional text not included in the text part of the message	See examples below.
Illustration	Illustration in the image—at least beyond use of a table and colors	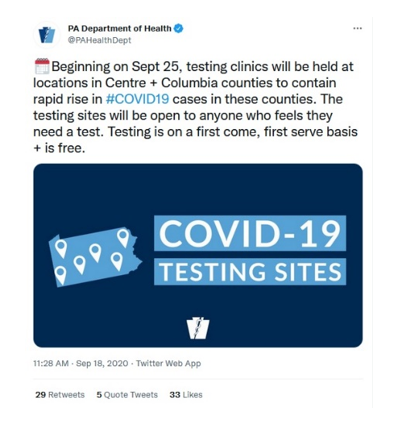
Photograph	Photograph of a person, object, or scene	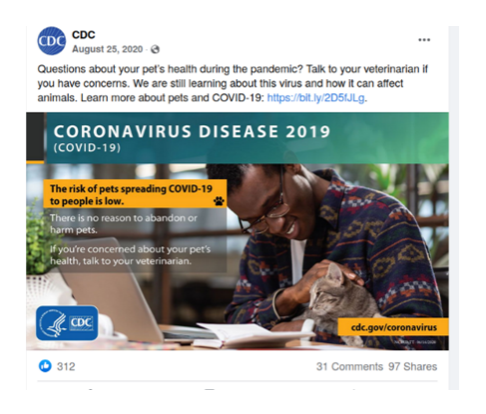
Infographic	Image that conveys data or illustrated directives (overrides illustration)	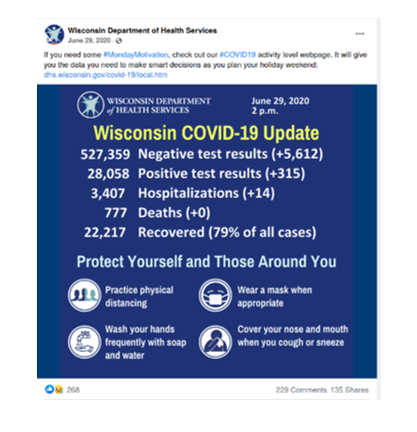
Video	A video embedded in the message	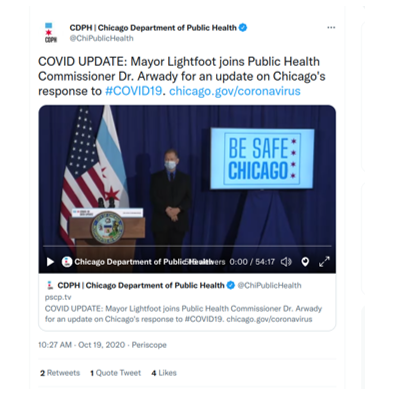

### Content Analysis

The content analysis consisted of manual binary coding for the presence or lack of each element in a post. As the definition of the categories became apparent, the nature of some definitions made some categories mutually exclusive, especially within each textual or media dimension. For example, a question is, by definition, not a *representative* and not an *expressive*. These coding rules are summarized in Tables 1 and 2 and are further detailed in Multimedia Appendix 2.

A random training sample of 150 posts (75, 50%, from Twitter and 75, 50%, from Facebook) was first retrieved for training and category development. Using these 150 posts, during training, 3 authors updated and defined the message categories. Once this training was accomplished, the 3 authors independently began coding a 20% subsample of the *sample data set*, where at least 2 coders double-coded the same post to calculate the Cohen κ statistic of interrater reliability (IRR).

After obtaining IRR measures, the coders discussed the results. At this point, the results were not perfect and discrepancies in coding existed and needed to be reconciled. In particular, there were issues with the *representative* and *request* speech functions, the *external speaker*, and some of the *rhetorical* dimensions. For example, it was not clear whether a slogan on an image, such as “COVID-19 news update,” was to be considered a *representative* sentence. We ultimately agreed on the definitions as shown in Multimedia Appendix 2, but IRR results were ultimately not perfect for all categories. The κ values are provided in Multimedia Appendix 1. After the IRR analyses, we discussed issues identifying the categories and then better defined and narrowed the rules for final coding of the data. In the cases that discrepancies existed across coders, and categories were revised, we re-examined the data based on the revised definitions and obtained agreement among coders. We then set out to code the remaining data. Each coder independently coded approximately 450 posts, producing a final sample data set of 1677 posts for statistical analysis.

### Statistical Analyses

To address our first RQ, we calculated the distribution of each message element on Twitter and Facebook and then compared this total across platforms via an independent 2-sample Z-test of proportions, where the null hypotheses assumed that the proportion of each message element is equal on both platforms. Although Z-tests expect normal distributions, and social media phenomena are notoriously not normally distributed, given the relatively large sample of most message elements, we found it reasonable to apply the Z-tests [44].

To address our second RQ, we operationalized audience engagement as normalized frequencies of likes and shares. Other studies have used the CTR to measure audience engagement [13], seemingly nonnormalized tweet counts [15], and regression models where follower count, and other dimensions, are controlled for [45]. The CTR measure used by Reuter et al [13] was not possible for our study since we could not have access to message clicks or actual message views (the total_views field provided by the Facebook API was not reliable and contained missing data; no such measure was provided by the Twitter API). Our approach is simpler than the regression models, but given the focus on a single issue, the random sampling of data across agencies and time, and normalized measure of likes and shares based on an agency’s follower count, our approach provides a robust and easy-to-interpret method to test the association between message features and audience engagement.

We calculated a measure of normalized likes (NL_m_) as the number of likes of each message “m,” divided by the follower count of the account that posted the message. NL_m_ is the percentage of the agency’s follower count that liked the message. Although Facebook includes additional positive and negative measures of audience engagement—namely *love*, *care*, *ha-ha*, *wow*, *sad*, and *angry*—these were not included as part of the NL_m_ measure to make it more comparable with the single *like* feature of Twitter. Although we considered and analyzed the more negative measures of Facebook sentiment, namely sad and angry, these overly complicated the research and ultimately seemed out of scope, since our aim was to compare Facebook and Twitter elements. This study thus focused on only likes and shares on Facebook and Twitter, both of which are types of *positive engagement.* Generally, in this study, engagement refers to “liking” or “sharing” a message.

Similar to normalized likes, we created a measure of normalized shares (NS_m_) of each message “m.” The NS_m_ measure, compared to likes, can be more directly considered a *diffusion rate* [46] or *retransmission rate* [7] of a message (or message elements), since it is a direct share by the user to its network. Although messages are not only liked or shared by the followers of an account, the size of an account’s followers largely influences the total engagement with posts of that account [47]. Equations of these normalized like and normalized share measures are provided in Multimedia Appendix 3.

For every message element, we then computed the *mean NS* and *mean NL* of all messages that contained the element, and of all messages that did not contain it, and compared these 2 groups via a 2-tailed independent-samples Wilcoxon-Mann-Whitney (WMW) test, given the skewness of the data and as similar studies have approached it [15]. We considered and discussed *P*≤.05 as statistically significant.

## Results

### Data Set Details

Table 3 shows descriptive statistics for the final sample data set in relation to the population data set of COVID-19–related posts. The list of agency accounts in the sample are in Multimedia Appendix 1. As shown in Table 3, local, state, and federal agencies made a comparable number of Facebook and Twitter posts (these measures do not include shares or retweets). In general, per account, state agencies were more active in posting than local and federal agencies. For example, on Facebook—based on population statistics—state accounts made 712 posts per account (34,930 total posts by 49 accounts), whereas local accounts made 377 posts per account (14,356 total posts by 38 accounts), and federal accounts made 359 posts per account (3592 total posts by 10 accounts). Results were relatively similar for Twitter.

**Figure 1:**
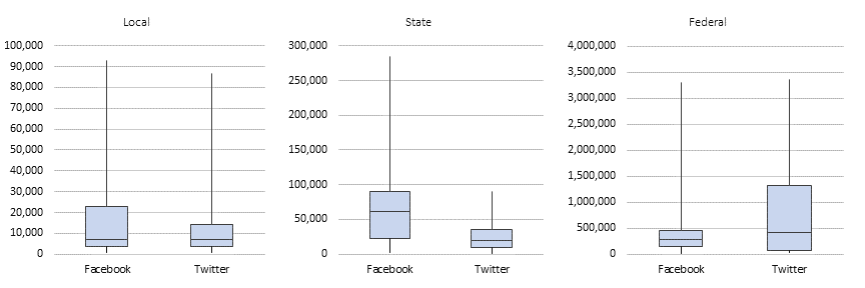
Box plot of IQRs of followers per account across agency levels and platforms.

Figure 1 shows the mean and IQR of account followers, separated for local, federal, and agency accounts (based on the sample data set). There were strong variations across local, state, and federal agencies in the distribution of followers and platforms. Not surprisingly, federal agency accounts had the most followers, and state agencies had more followers than local agencies, on average. Federal agencies were more popular (ie, had more followers) on Twitter, whereas state agencies were more popular on Facebook. Local agencies were similarly popular on Facebook and Twitter. Generally, there is great variation in the top quartile of the distribution. Detailed numbers for this box plot can be found in Multimedia Appendix 3.

**Table 3:** Statistics^a^ for the sample data set as a percentage of the population of COVID-19 posts in 2020.

	Local, n/N (%)	State, n/N (%)	Federal, n/N (%)	All, n/N (%)
Facebook accounts	32/38 (84.0)	48/49 (98.0)	9/10 (90.0)	89/97 (92.0)
Twitter accounts	29/33 (88.0)	45/48 (94.0)	9/11 (82.0)	83/92 (90.0)
Facebook total posts	231/14,356 (1.6)	560/34,930 (1.6)	60/3592 (1.7)	851/52,878 (1.6)
Twitter total posts	262/15,421 (1.7)	482/27,866 (1.7)	82/4620 (1.8)	826/47,907 (1.7)

^a^Statistics are for the final sample data set used in content and statistical analyses in relation to the population data set of all COVID-19–related posts from all accounts identified in 2020.

### Platform Effects on Message Design

Table 4 shows the total count of each message element in the coded sample data set as the number of posts in which the element appeared, separately for Facebook and Twitter. Table 4 also provides results from a 2-tailed Z-test that compares whether the proportions are equal across platforms. Results showed that most features are used to a similar extent across platforms. These results provide some validity for the notion that these message features are indeed part of public health and risk communication on social media more broadly. However, we also found some statistically significant differences across the 2 sites. A positive Z-score indicates higher use on Twitter; a negative score indicates higher use on Facebook.

**Figure 2:**
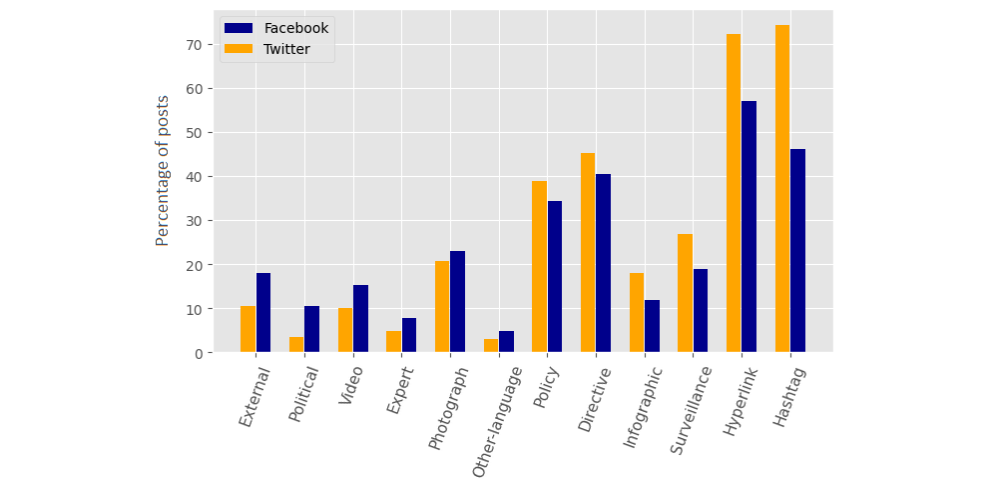
Elements used significantly more on Facebook and significantly more on Twitter.

Figure 2 shows the message elements used significantly more or less on Facebook or Twitter, relative to each other, the bars identifying the percentage of posts in which each message element appeared. *External*, *political*, and *expert* actors, along with *video*, *photograph*, and *other language*, were the features more frequently used in Facebook posts compared to Twitter posts. *Policy*, *directive*, *infographic*, *surveillance*, *hyperlink,* and *hashtag* features were used more frequently on Twitter compared to Facebook. *Personality* and *positive* framing features were not included in Figure 2 due to the low sample size. However, *policy* was included in the graph, although at the significance boundary.

**Table 4:** Message design elements across Facebook (n=851) and Twitter (n=826) posts.

Message element			Facebook, n (%)	Twitter, n (%)	Z-score	*P* value
**Speech function**						
	Representative	755 (88.7)		722 (87.4)	–0.83	.41
	Directive	344 (40.4)		374 (45.2)	2.01	.04
	Question	107 (12.5)		96 (11.6)	–0.60	.55
	Expressive	79 (9.2)		77 (9.3)	0.03	.98
	Request	28 (3.2)		38 (4.6)	1.40	.17
**Topic**						
	Protection	391 (45.9)		395 (47.8)	0.77	.44
	Policy	292 (34.3)		321 (38.8)	1.93	.05
	Surveillance	160 (18.8)		222 (26.8)	3.94	<.001
	Science	73 (8.5)		78 (9.4)	0.62	.53
	Emergent	39 (4.5)		26 (3.1)	–1.52	.13
**Resource type**						
	Interactive	192 (22.5)		175 (21.1)	–0.68	.49
	Material	112 (13.1)		112 (13.5)	0.24	.81
	Corrective	11 (1.2)		12 (1.4)	0.28	.78
**Focus and audience**						
	Group	85 (9.9)		113 (13.6)	2.34	.02
	Secondary	73 (8.5)		59 (7.1)	–1.09	.27
	Other language	42 (4.9)		25 (3.0)	–1.99	.04
**Speaker**						
	External	153 (17.9)		86 (10.4)	–4.43	<.001
	Political	89 (10.4)		28 (3.3)	–5.68	<.001
	Expert	66 (7.7)		39 (4.7)	–2.56	.01
	Personality	17 (1.9)		5 (0.6)	–2.51	.01
**Rhetorical**						
	Collective	123 (14.4)		105 (12.7)	–1.04	.30
	Emphasis	103 (12.1)		81 (9.8)	–1.50	.13
	Positive	12 (1.4)		23 (2.7)	1.97	.05
	Metaphor	5 (0.5)		2 (0.2)	–1.10	.27
**Media**						
	Hyperlink	485 (56.9)		597 (72.2)	6.54	<.001
	Hashtag	392 (46.0)		613 (74.2)	11.76	<.001
	Text-in-image	387 (45.4)		343 (41.5)	–1.63	.10
	Illustration	235 (27.6)		258 (31.2)	1.63	.10
	Photograph	196 (23.0)		170 (20.5)	–1.22	.22
	Infographic	101 (11.8)		149 (18.0)	3.55	<.001
	Video	130 (15.2)		83 (10.0)	–3.21	<.001

### Platform Effects on Audience Engagement

Tables 5 and 6 show audience engagement with messages containing each specific feature compared to those without the feature, calculated separately for Facebook and Twitter as normalized likes and normalized shares. In general, Facebook had higher engagement of users compared to Twitter. In addition, Facebook users used shares more frequently than likes, while Twitter users liked more frequently than they shared. Facebook posts, on average, were liked by 0.19% of account followers, whereas on Twitter, on average, posts were liked by 0.08% of account followers, a difference of 2.25 times higher for Facebook likes. Regarding sharing, Facebook posts, on average, were shared by 0.22% of account followers, whereas on Twitter, on average, posts were shared by 0.05% of account followers, which is more than a 4.4 times difference. However, these engagement measures do not include other forms of engagement on Facebook (eg, *love*, *care*), as previously discussed under Methods.

Table 5 provides the mean normalized likes of all messages with the feature compared to those without it, along with *P* values for the WMW test comparing these 2 sets. For example, in the Facebook sample, on average, 0.16% of the (count of the) account’s followers liked the message that contained a *representative*, whereas 0.26% liked the messages that did not contain a *representative*. Therefore, on Facebook, messages that did not contain a *representative* were liked more than messages that did. However, this was not a statistically significant difference (*P*=.22). On Twitter, however, on average, 0.08% of the account’s followers liked messages that contained a *representative* and 0.05% liked messages that did not contain it, which was a significant difference (*P*<.001).

Table 6 provides the mean normalized shares of all messages with the feature and those without it, along with *P* values from the WMW test comparing differences between them. Results here can be similarly interpreted as the results in Table 5.

**Figure 3:**
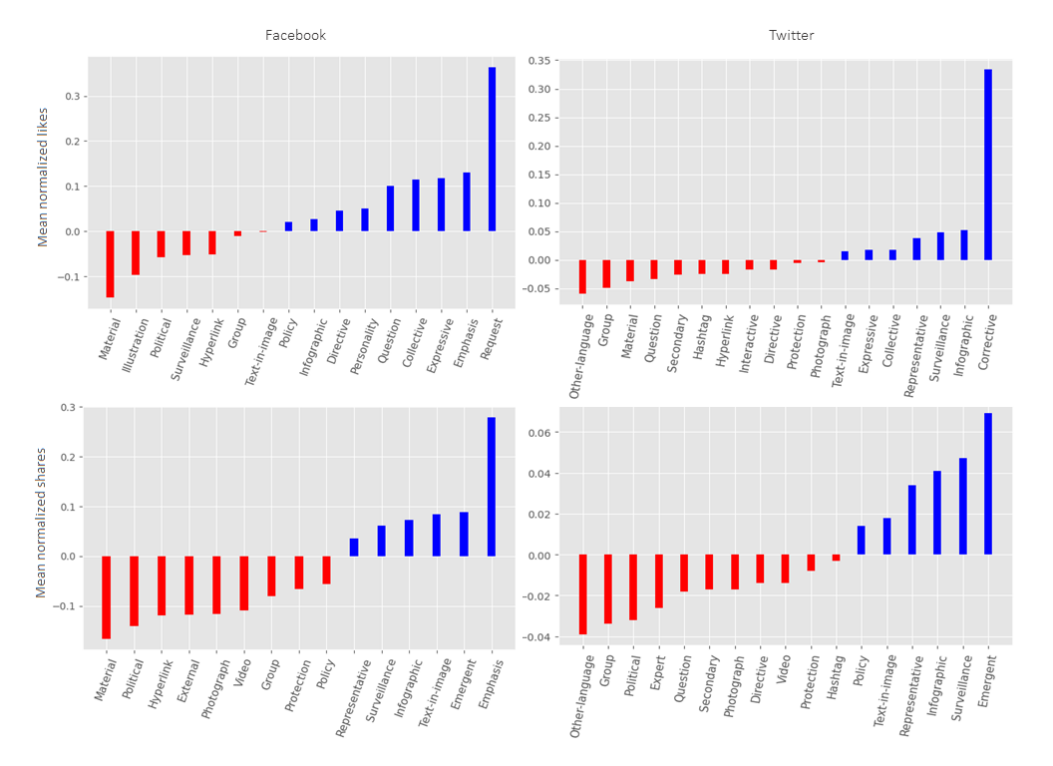
Significant changes in likes and shares associated with the inclusion of message element. The blue bars refer to increases and the red bars to decreases in mean normalized likes and mean normalized shares associated with the inclusion of the message element.

Figure 3 shows the message elements from Tables 5 and 6 that had a significant association with an increase or decrease in normalized likes and shares. Figure 3 shows the percentage points in the increase/decrease associated with the inclusion of the message element. *Expressives* and the use of a *collective* frame in messages were associated with more likes across both platforms. *Surveillance* information as well as *infographics* were also associated with more likes and shares across Facebook and Twitter. References to *material* resources, surprisingly, were generally associated with fewer likes and shares on both platforms. We speculate this may be due to the repeated posts about testing and vaccine sites coded under *material*. Although *political* figures were more present on Facebook compared to Twitter, they were associated with less engagement on both platforms, especially Facebook. *Requests* were particularly popular on Facebook but not significant on Twitter. *Correctives* and *policy information* were associated with higher engagement on Twitter but less so or not significantly on Facebook.

**Table 5:** Mean percentage of account followers that liked messages with and without specific elements.

Message element			Facebook							Twitter				
			With feature		Without feature		*P* value^a^		With feature			Without feature		*P* value^a^
**Speech function**														
	Representative	0.16		0.26		.22		0.08			0.05		<.001	
	Directive	0.20		0.15		.01		0.07			0.09		<.001	
	Question	0.26		0.16		.04		0.05			0.08		<.001	
	Expressive	0.28		0.16		<.001		0.10			0.08		<.001	
	Request	0.52		0.16		.05		0.06			0.08		.32	
**Topic**														
	Protection	0.18		0.17		.43		0.08			0.08		.02	
	Policy	0.19		0.17		.03		0.09			0.07		.20	
	Surveillance	0.13		0.18		.02		0.12			0.07		<.001	
	Science	0.14		0.18		.41		0.05			0.08		.08	
	Emergent	0.14		0.17		.26		0.25			0.07		.06	
**Resource type**														
	Interactive	0.17		0.17		.20		0.07			0.08		.04	
	Material	0.05		0.19		<.001		0.05			0.08		<.001	
	Corrective	0.18		0.17		.49		0.41			0.07		.03	
**Focus and audience**														
	Group	0.16		0.17		<.001		0.04			0.09		<.001	
	Secondary	0.13		0.18		.13		0.06			0.08		.01	
	Other language	0.10		0.18		.07		0.02			0.08		<.001	
**Speaker**														
	External	0.13		0.18		.07		0.06			0.08		.13	
	Political	0.12		0.18		.01		0.06			0.08		.08	
	Expert	0.17		0.17		.06		0.06			0.08		.42	
	Personality	0.22		0.17		.01		0.06			0.08		.30	
**Rhetorical**														
	Collective	0.27		0.16		<.001		0.10			0.08		.004	
	Emphasis	0.29		0.16		.004		0.08			0.08		.10	
	Positive	0.41		0.17		.12		0.10			0.08		.43	
	Metaphor	0.41		0.17		.09		0.02			0.08		.26	
**Media**														
	Hyperlink	0.15		0.20		<.001		0.07			0.10		<.001	
	Hashtag	0.19		0.16		.32		0.07			0.10		.01	
	Text-in-image	0.17		0.17		.01		0.09			0.07		.002	
	Illustration	0.10		0.20		.03		0.06			0.09		.12	
	Photograph	0.21		0.16		.08		0.07			0.08		<.001	
	Infographic	0.20		0.17		<.001		0.12			0.07		<.001	
	Video	0.21		0.17		.07		0.07			0.08		.09	

^a^*P* values refer to the Wilcoxon-Mann-Whitney test of comparing the mean normalized likes for posts containing the feature with those not containing it, separately for Facebook and Twitter.

**Table 6:** Mean percentage of account followers that shared messages with and without specific features.

Message element		Facebook			Twitter		
		With feature	Without feature	*P* value^a^	With feature	Without feature	*P* value^a^
**Speech function**							
	Representative	0.20	0.16	.01	0.06	0.03	<.001
	Directive	0.17	0.21	.27	0.05	0.06	<.001
	Question	0.22	0.19	.10	0.04	0.06	.003
	Expressive	0.29	0.19	.06	0.07	0.05	.06
	Request	0.53	0.18	.18	0.05	0.06	.39
**Topic**							
	Protection	0.16	0.23	.03	0.05	0.06	.002
	Policy	0.16	0.21	.004	0.06	0.05	.04
	Surveillance	0.25	0.18	<.001	0.09	0.04	<.001
	Science	0.15	0.20	.36	0.04	0.06	.05
	Emergent	0.28	0.19	.04	0.12	0.05	.03
**Resource type**							
	Interactive	0.30	0.17	.34	0.05	0.06	.35
	Material	0.05	0.22	<.001	0.04	0.06	.45
	Corrective	0.18	0.20	.29	0.18	0.05	.19
**Focus and audience**							
	Group	0.12	0.20	.001	0.03	0.06	<.001
	Secondary	0.33	0.18	.19	0.04	0.06	.01
	Other language	0.09	0.20	.16	0.02	0.06	.001
**Speaker**							
	External	0.10	0.22	.04	0.05	0.06	.18
	Political	0.07	0.21	<.001	0.02	0.06	.01
	Expert	0.07	0.21	.26	0.03	0.06	.02
	Personality	0.08	0.20	.31	0.03	0.06	.21
**Rhetorical**							
	Collective	0.20	0.19	.07	0.07	0.05	.28
	Emphasis	0.44	0.16	.04	0.06	0.05	.31
	Positive	0.22	0.20	.34	0.05	0.06	.40
	Metaphor	1.63	0.19	.27	0.01	0.06	.14
**Media**							
	Hyperlink	0.14	0.26	.003	0.05	0.06	.08
	Hashtag	0.26	0.14	.37	0.05	0.06	.04
	Text-in-image	0.24	0.16	<.001	0.07	0.05	<.001
	Illustration	0.15	0.21	.40	0.05	0.06	.13
	Photograph	0.11	0.22	<.001	0.04	0.06	<.001
	Infographic	0.26	0.19	<.001	0.09	0.05	<.001
	Video	0.10	0.21	.01	0.04	0.06	.01

^a^*P* values refer to the Wilcoxon-Mann-Whitney test of comparing the mean normalized shares for posts containing the feature with those not containing it, separately for Facebook and Twitter.

## Discussion

### Principal Findings

This study analyzed 1677 COVID-19–related posts on Facebook and Twitter, by public health agencies across the United States in 2020, and found differences and similarities in the overall use and popularity of these sites in terms of message design elements and audience engagement. Our results show that Facebook posts received 2.25 times more likes and 4.4 times more shares, in general, than posts on Twitter. However, within each platform, messages received more shares than likes within Facebook—as a percentage of account followers that liked or shared the message—whereas on Twitter, measures were more liked than shared.

Our results show that messages on Twitter, compared to Facebook, are significantly more focused on *surveillance information* (eg, data and statistics about the threat), *policy information*, *infographics*, and *hyperlinks*. Moreover, federal agencies are more active and more popular on Twitter compared to Facebook, whereas local and state agencies are more active or more popular on Facebook. We also observe that messages on Facebook, compared to Twitter, have significantly more references to *political figures*, public health *experts*, and (nonpolitical) *personalities* (eg, personal stories or local celebrities) as speakers in the messages. From this, we may conclude a type of *data and policy* orientation for Twitter and a *local and personal orientation* for Facebook.

We observed that data (eg, *infographics*, *surveillance* data) and *policy* information had significant positive associations with audience engagement on Twitter but not at all or not as much on Facebook, further suggesting this data and policy characterization for Twitter. Although Facebook was the platform where political figures and health experts were more highlighted as speakers in the messages, this personalization was generally not associated with higher engagement on both sites. However, we observe that *photographs*, which are often of people, and rhetorical elements, such as a *collective* framing (eg, “we are in this together”), *positive* framing (eg, “we are trying our best”), and *emphasis* (eg, exclamation points), which may trigger sentiment and personal connection, received more or significantly more audience engagement on Facebook but not as much or not at all on Twitter. This further suggests the local and personal orientation for Facebook.

The distribution of message design elements is largely similar across both platforms, suggesting consistency in public health messaging, but with some significant differences between the 2 social media sites studied. Results also show significant associations between message elements and audience engagement, with some expected and surprising differences across platforms. In general—for this health and risk communication scenario—we may thus suggest that Twitter has more of a data and policy orientation, whereas Facebook has more of a local and personal orientation on the content, which largely follows the literature on social media affordances.

### Integration With Existing Literature

Previous studies have examined the characteristics of Facebook in relation to Twitter as 2 of the major social media sites in the United States and in the world today. Generally, studies support the notion that Twitter is more of a “news media” [22,36] for “information dissemination” [38] and for being “quickly informed” [39], while Facebook is more for “shared identities,” “photographs” [24], and “social interaction” [39], being associated more with bonding social capital [22]. This distinction between Twitter and Facebook is usually explained as the specific affordances of each site [13,25], which may be related to some of its technical features, such as the more open unidirectional networks of Twitter compared to the bidirectional networks of Facebook [38]. Studies also suggest that certain technical features of a site (eg, focus on visual imagery) may lead to an overall higher audience engagement [13,22].

In this study, we did not analyze whether certain platform features caused the use of specific message elements or whether certain message features caused more or less engagement. However, our results generally support the existing literature that suggests that Facebook, while bigger and more popular across the US adult population, has more of a local and personal orientation, associated with close social interactions. Twitter, in contrast, is both a more active and a popular site for federal agencies, compared to local and state agencies, and both the content and engagement on Twitter point to more of a data and policy orientation. Ultimately, we observe great similarities in message elements and audience engagement across Facebook and Twitter, suggesting a standardization of social media policies and practices across agencies and platforms, and also similarities in user engagement on both Facebook and Twitter.

### Contributions to Health Communication Policy

This study provides some evidence for policy recommendations on social media health communication strategies. These recommendations are based on the results of this study, which is focused on COVID-19 communication during the beginning and multiple waves of the pandemic in 2020. Public health agencies and further research need to assess whether these are valid for broader contexts as well.

#### Recommendation 1

For public health agencies using Facebook, we recommend caution when using political figures and external experts on their messages and instead highlight nonpolitical or nongovernment personalities, such as local celebrities or ordinary individuals who have a special story to tell. We also see an opportunity for greater or at least continued use of emotional expressions on messages and the use of collective frames to generate greater positive engagement.

Our results show that messages on Facebook, compared to Twitter, are significantly more focused on highlighting political figures, as well as internal and external experts. However, political figures and external experts were generally associated with less engagement on Facebook. Personalities, including celebrities or ordinary people (eg, an authentic post of a child from the community), were significantly associated with greater engagement on Facebook but were present in few posts (2%) on Facebook. Ultimately, the use of expressives (ie, expressing emotions) and collective frames (eg, using collective pronouns and focusing on collective issues) were particularly well engaged with on Facebook.

#### Recommendation 2

For public health agencies using Twitter, we recommend caution on the use of hyperlinks and hashtags on Twitter messages if the goal is to increase message likes and overall message diffusion, but continued use of surveillance information and infographics is recommended. Moreover, we recommend a greater focus on messages containing emergent issues (eg, emergency or timely information), and the use of correctives to address misinformation, because these were both not prevalent but were associated with greater positive engagement.

Our results show that messages on Twitter, compared to Facebook, are significantly more focused on policy and surveillance information and include significantly more hyperlinks and hashtags compared to messages on Facebook. Since the hashtag is a textual construction first popularized on Twitter, this is not surprising. However, both hashtags and hyperlinks were generally associated with less engagement on Twitter. Surveillance information and infographics, however, were generally associated with greater engagement on Twitter. Emergent issues, and correctives, were particularly well engaged with on Twitter. However, correctives were included in a minority of tweets (1.4%). Given that social media is part of a misinformation crisis [48], or *infodemic* [49,50], it is important to consider how public health agencies are addressing misinformation on these environments.

#### Recommendation 3

For public health agencies using both platforms, we recommend careful use of images in their messages, including photographs, illustrations, and videos, as these were all media types associated with less engagement across both platforms. However, including text-in-image is a reasonable recommendation, since these were associated with greater engagement across platforms.

In general, our results show that not all types of images are similarly engaged with. On both platforms, photographs were significantly associated with fewer shares, whereas infographics were generally associated with greater shares and likes. Although illustrations were associated with fewer likes and shares on both platforms, this negative impact was only significant for Facebook likes. Infographics about the pandemic were associated with higher engagement on both platforms, but they were also largely prevalent. Therefore, the amount of use of these features in this context is likely sufficient. Lastly, text-in-image was generally associated with greater likes and shares on Twitter and greater sharing on Facebook, highlighting the importance of textual and semantic content along with visual content.

### Limitations and Future Work

This study intended to show how public health agencies construct their messages across Facebook and Twitter and how users respond to these messages similarly or differently across platforms. To control for aspects of the message topic, we only focused on COVID-19–related messages. COVID-19 is also a major health and risk issue and one that we could expect public health agencies in the country to be communicating about in 2020. However, the focus on COVID-19 puts a limitation on the extent to which we can generalize the findings to health and risk communication more broadly. Moreover, the statistical tests used could be improved with a regression model that assesses and controls for other variables on audience engagement. Nevertheless, our random sampling technique, over multiple kinds of agencies and an entire year, helps us generalize and have confidence in the results.

Health communicators should consider that social media algorithms themselves are problematic as they lead to echo chamber effects [35] and are biased toward hyperactive users [51]. Audience engagement on social media itself should thus be considered with care. The literature generally points to social media engagement as being driven by high emotional content [52], out-group animosity [53], and fear-arousing sensationalism [54]. Simply acquiring more engagement is thus not always appropriate for health and risk communicators. Moreover, there is a chance that social media in government may be used for political purposes [55,56]. Future studies may thus advance this work by examining the *quality* of engagement across platforms, political issues in public health communication, and examining the nature of the comments to public health messages.

There were few posts with personalities featured on Facebook (17/851, 1.9%) and Twitter (5/826, 0.6%) posts. We could thus not properly assess the impact of this message element on engagement. However, celebrities and personal stories can positively influence health behavior and may be further studied in this context [54,57]. In addition, analyses of fear appeals, distinctions between more or less informative (or scientific) messages, or the use of storytelling, could have improved this study. Some message features need better definition to increase reliability, including representatives and requests. The category of representatives and its results here should be considered with caution, since it is the broadest category of the framework and had a low κ. In all, future research may gain from refining the framework categories, further examining the use of celebrities or personal stories, and the relationship between fear-appeals or other rhetorical strategies on different levels and qualities of user engagement.

### Conclusion

In general, we find a data and policy orientation for Twitter messages and users and a local and personal orientation for Facebook, although also many similarities across both platforms. Message elements that impact engagement are similar across both platforms but with some notable distinctions. This study provides novel evidence for differences in COVID-19 public health messaging on social media, advancing health communication research and recommendations for health and risk communication strategies.

## Appendix

Multimedia Appendix 1 Sampled accounts and κ values.

Multimedia Appendix 2 Detailed framework description and coding rules.

Multimedia Appendix 3 Sample statistics and analyses.

## Abbreviations

API: application programming interface
CTR: click-through rate
IRR: interrater reliability
RQ: research question
WMW: Wilcoxon-Mann-Whitney
