# Supplementary material for: Platform Effects on Public Health Communication: A Comparative and National Study of Message Design and Audience Engagement Across Twitter and Facebook
Source: JMIR Infodemiology. 2022 Dec 20;2(2):e40198. doi: 10.2196/40198 (PMC9773105; doi:10.2196/40198)
Supplement: Multimedia Appendix 1 [file infodemiology_v2i2e40198_app1.docx]

**Appendix I**

List of agencies and specific accounts sampled in this study (accounts n=174, agencies n=95).

| **Handle** | **Platform** | **Agency Name** |
| --- | --- | --- |
| ***Federal accounts (n=19) Unique agencies (n=9)*** | | |
| ahrqnews | Twitter | Agency for Healthcare Research and Quality (AHRQ) |
| ahrq.gov | Facebook | Agency for Healthcare Research and Quality (AHRQ) |
| cdcemergency | Twitter | CDC Emergency Preparedness and Response |
| cdcemergency | Facebook | CDC Emergency Preparedness and Response |
| cdcgov | Twitter | Centers for Disease Control and Prevention |
| cdc | Facebook | Centers for Disease Control and Prevention |
| niaid.nih | Facebook | National Institute of Allergy and Infectious Diseases (NIAID) |
| niaidnews | Twitter | National Institute of Allergy and Infectious Diseases (NIAID) |
| nih.gov | Facebook | National Institutes of Health (NIH) |
| nih | Twitter | National Institutes of Health (NIH) |
| nationallibraryofmedicine | Facebook | National Library of Medicine (NLM) |
| nlm_nih | Twitter | National Library of Medicine (NLM) |
| niosh | Facebook | NIOSH National Institute for Occupational Safety and Health |
| niosh | Twitter | NIOSH National Institute for Occupational Safety and Health |
| hhsgov | Twitter | U.S. Department of Health and Human Services |
| hhs | Facebook | U.S. Department of Health and Human Services |
| fda | Facebook | U.S. Food and Drug Administration |
| us_fda | Twitter | U.S. Food and Drug Administration |
| hrsagov | Twitter | U.S. Health Resources and Services Administration |

| ***State accounts (n=99) Unique agencies (n=52)*** | | |
| --- | --- | --- |
| alabamapublichealth | Facebook | Alabama Public Health |
| alpublichealth | Twitter | Alabama Public Health |
| alaska.dhss | Facebook | Alaska Health and Social Services |
| alaska_dhss | Twitter | Alaska Health and Social Services |
| azdhs | Twitter | Arizona Department of Health Services |
| azdhs | Facebook | Arizona Department of Health Services |
| arhealthdept | Facebook | Arkansas Department of Health |
| adhpio | Twitter | Arkansas Department of Health |
| capublichealth | Twitter | California Department of Public Health |
| capublichealth | Facebook | California Department of Public Health |
| cdphe | Twitter | Colorado Department of Public Health & Environment |
| cdphe | Facebook | Colorado Department of Public Health and Environment |
| ctdph | Twitter | Connecticut Department of Public Health |
| ctpublichealth | Facebook | Connecticut Department of Public Health |
| _dchealth | Twitter | DC Health |
| dchealth.dc.gov | Facebook | DC Health |
| delawaredhss | Facebook | Delaware Department of Health and Social Services (DHSS) |
| delaware_dhss | Twitter | Delaware Department of Health and Social Services (DHSS) |
| depublichealth | Facebook | Delaware Department of Health and Social Services (DHSS) |
| fldepartmentofhealth | Facebook | Florida Department of Health (DOH) |
| healthyfla | Twitter | Florida Department of Health (DOH) |
| gadph | Facebook | Georgia Department of Public Health |
| gadph | Facebook | Georgia Department of Public Health |
| hawaiidoh | Twitter | Hawaii State Department of Health |
| hawaiidoh | Facebook | Hawaii State Department of Health |
| idhw | Twitter | Idaho Department of Health and Welfare |
| idahohealthandwelfare | Facebook | Idaho Department of Health and Welfare |
| idph.illinois | Facebook | Illinois Department of Public Health (IDPH) |
| idph | Twitter | Illinois Department of Public Health (IDPH) |
| statehealthin | Facebook | Indiana Department of Health |
| statehealthin | Twitter | Indiana Department of Health |
| iapublichealth | Twitter | Iowa Department of Public Health |
| iowadepartmentofpublichealth | Facebook | Iowa Department of Public Health |
| kdhenews | Facebook | Kansas Department of Health and Environment |
| kdhe | Twitter | Kansas Department of Health and Environment |
| kyhealthalerts | Twitter | Kentucky Department for Public Health |
| kypublichealth | Facebook | Kentucky Department for Public Health |
| ladepthealth | Facebook | Louisiana Department of Health |
| ladepthealth | Twitter | Louisiana Department of Health |
| mdhealthdept | Facebook | Maryland Department of Health |
| mdhealthdept | Twitter | Maryland Department of Health |
| massdph | Twitter | Massachusetts Department of Public Health |
| michiganhhs | Twitter | Michigan Department of Health and Human Services |
| michiganhhs | Facebook | Michigan Department of Health and Human Services |
| mnpoisoncenter | Twitter | Minnesota Department of Health |
| mnhealth | Facebook | Minnesota Department of Health |
| healthyms | Facebook | Mississippi State Department of Health |
| msdh | Twitter | Mississippi State Department of Health |
| healthylivingmo | Twitter | Missouri Department of Health and Senior Services |
| healthylivingmo | Facebook | Missouri Department of Health and Senior Services |
| mtdphhs | Facebook | Montana Department of Public Health and Human Services |
| ncdhhs | Facebook | NC Department of Health and Human Services |
| ncdhhs | Twitter | NC Department of Health and Human Services |
| nedhhs | Facebook | Nebraska Department of Health and Human Services |
| nedhhs | Twitter | Nebraska Department of Health and Human Services |
| nvhealthandhumanservices | Facebook | Nevada Department of Health and Human Services |
| nhdhhspio | Twitter | New Hampshire Department of Health and Human Services |
| nhdepartmentofhealthandhumanservices | Facebook | New Hampshire Department of Health and Human Services |
| njdeptofhealth | Facebook | New Jersey Department of Health |
| njdeptofhealth | Twitter | New Jersey Department of Health |
| nmdoh | Facebook | New Mexico Department of Health |
| nmdoh | Twitter | New Mexico Department of Health |
| nolahealthdept | Twitter | New Orleans Health Department |
| nolahealthdept | Facebook | New Orleans Health Department |
| nddoh | Twitter | North Dakota Department of Health |
| ndhealth | Facebook | North Dakota Department of Health |
| healthnygov | Twitter | NYSDOH - New York State Health Department |
| nysdoh | Facebook | NYSDOH - New York State Health Department |
| ohdeptofhealth | Twitter | Ohio Department of Health |
| ohdeptofhealth | Facebook | Ohio Department of Health |
| healthyoklahoma | Twitter | Oklahoma State Department of Health |
| oklahomahealth | Facebook | Oklahoma State Department of Health |
| ohaoregon | Twitter | OR Health Authority |
| pennsylvaniadepartmentofhealth | Facebook | Pennsylvania Department of Health |
| pahealthdept | Twitter | Pennsylvania Department of Health |
| healthri | Facebook | Rhode Island Department of Health |
| rihealth | Twitter | Rhode Island Department of Health |
| scdhec | Facebook | South Carolina Department of Health & Environmental Control |
| scdhec | Twitter | South Carolina Department of Health & Environmental Control |
| sddoh | Twitter | South Dakota Department of Health |
| sdhealthdepartment | Facebook | South Dakota Department of Health |
| southernnevadahealthdistrict | Facebook | Southern Nevada Health District |
| snhdinfo | Twitter | Southern Nevada Health District |
| tndeptofhealth | Twitter | Tennessee Department of Health |
| tndeptofhealth | Facebook | Tennessee Department of Health |
| texasdshs | Twitter | Texas Department of State Health Services |
| texasdshs | Facebook | Texas Department of State Health Services |
| utahdepofhealth | Facebook | Utah Department of Health |
| healthvermont | Twitter | Vermont Department of Health |
| healthvermont | Facebook | Vermont Department of Health |
| vdhgov | Facebook | Virginia Department of Health |
| vdhgov | Twitter | Virginia Department of Health |
| wadepthealth | Twitter | Washington State Department of Health |
| wadepthealth | Facebook | Washington State Department of Health |
| dhswi | Facebook | Wisconsin Department of Health Services |
| dhswi | Twitter | Wisconsin Department of Health Services |
| wv_dhhr | Twitter | WV Department of Health & Human Resources |
| wv.dhhr | Facebook | WV Department of Health & Human Resources |
| wdhphep | Facebook | Wyoming Department of Health - PHEP |

| ***Local accounts (n=56) Unique agencies (n=34)*** | | |
| --- | --- | --- |
| ancpublichealth | Twitter | Anchorage Health Department |
| ancpublichealth | Facebook | Anchorage Health Department |
| bmore_healthy | Twitter | Baltimore City Health Department |
| baltimorehealth | Facebook | Baltimore City Health Department |
| healthyboston | Twitter | Boston Public Health |
| cdhidaho | Facebook | Central District Health |
| cdhidaho | Twitter | Central District Health |
| Cheyenne-Laramie County Health Department | Facebook | Cheyenne-Laramie County Health Department |
| chipublichealth | Twitter | Chicago Department of Public Health |
| chicagopublichealth | Facebook | Chicago Department of Public Health |
| kcmohealthdept | Facebook | City of Kansas City, Mo., Health Department |
| kcmohealthdept | Twitter | City of Kansas City, Mo., Health Department |
| mkehealth | Facebook | City of Milwaukee Health Department |
| mkehealth | Twitter | City of Milwaukee Health Department |
| columbuspublichealth | Facebook | Columbus Public Health |
| ddphe | Twitter | Denver Public Health & Environment |
| dethealth | Twitter | Detroit Health Department |
| dethealth | Facebook | Detroit Health Department |
| healthdouglasco | Twitter | Douglas County Health Department |
| douglascountyhealth | Facebook | Douglas County Health Department |
| fargocasspublichealth | Facebook | Fargo Cass Public Health |
| fcph | Twitter | Fargo Cass Public Health |
| fc_publichealth | Twitter | Franklin County PH |
| houstonhealth | Facebook | Houston Health Department |
| houstonhealth | Twitter | Houston Health Department |
| indyophs | Facebook | Indianapolis Office of Public Health & Safety |
| jeffersoncountydepartmentofhealth | Facebook | Jefferson County Department of Health - AL |
| jeffcohealthmo | Twitter | Jefferson County Health Department - MO |
| jeffersoncountypublichealth | Facebook | Jefferson County Public Health - WA |
| kchealth1 | Twitter | Kanawha-Charleston Health Department |
| kchdwv | Facebook | Kanawha-Charleston Health Department |
| lapublichealth | Twitter | Los Angeles County Department of Public Health |
| lapublichealth | Facebook | Los Angeles County Department of Public Health |
| loumetrohealth | Twitter | Louisville Metro Dept. of Public Health & Wellness |
| loumetrohealth | Facebook | Louisville Metro Dept. of Public Health & Wellness |
| mcdph | Facebook | Maricopa County Public Health |
| maricopahealth | Twitter | Maricopa County Public Health |
| mecklenburgcounty | Facebook | Mecklenburg County Government |
| nashvillehealth | Twitter | Metro Nashville Public Health Department |
| mphdnashville | Facebook | Metro Nashville Public Health Department |
| multcohealth | Facebook | Multnomah County Health Department |
| multcohealth | Twitter | Multnomah County Health Department |
| newarkdhcw | Facebook | Newark Department of Health and Community Wellness |
| newarkdhcw | Twitter | Newark Department of Health and Community Wellness |
| nychealth | Facebook | NYC Department of Health and Mental Hygiene |
| nychealthy | Twitter | NYC Department of Health and Mental Hygiene |
| occhd | Facebook | OKC-County Health Department |
| okchealth | Twitter | OKC-County Health Department |
| phlpublichealth | Twitter | Philadelphia Department of Public Health |
| phillyhealth | Facebook | Philadelphia Department of Public Health |
| polkcohealth | Twitter | Polk County Health Department |
| polkcohealth | Facebook | Polk County Health Department |
| healthycommunitiespvd | Facebook | Providence Healthy Communities |
| kcpubhealth | Twitter | Public Health - Seattle & King County |
| kcpubhealth | Facebook | Public Health - Seattle & King County |
| saltlakehealth | Twitter | Salt Lake Health |

**Cohen’s Kappa Results**

Cohen’s kappa coefficients provide values between 0 and 1 for every item coded by two raters to assess the reliability of the category. Generally, levels below .41 are interpreted as weak; between .41 to .60 are weak to moderate; above .61 moderate to substantial; and between .81 to 1 as strong to almost perfect [1,2]. Cohen’s kappa values resulted from our study are provided below.

| **dimension** | **feature** | **mean kappa** |
| --- | --- | --- |
| Audience and Focus | other-language | 1.00 |
| Media | video | 0.99 |
| Media | photograph | 0.98 |
| Speaker | political | 0.97 |
| Topic | surveillance | 0.89 |
| Resource | material | 0.87 |
| Speech function | question | 0.85 |
| Speech function | directive | 0.84 |
| Media | text-in-image | 0.84 |
| Media | infographic | 0.84 |
| Media | illustration | 0.83 |
| Resource | corrective | 0.83 |
| Topic | protection | 0.79 |
| Speaker | personality | 0.79 |
| Topic | emergent | 0.75 |
| Topic | science | 0.75 |
| Rhetoric | metaphor | 0.75 |
| Speech function | expressive | 0.75 |
| Speaker | expert | 0.73 |
| Topic | policy | 0.72 |
| Resource | interactive | 0.71 |
| Audience and Focus | secondary | 0.70 |
| Rhetoric | collective | 0.68 |
| Rhetoric | positive | 0.66 |
| Audience and Focus | group | 0.65 |
| Rhetoric | emphasis | 0.62 |
| Speech function | request | 0.61 |
| Speaker | external | 0.60 |
| Speech function | representative | 0.60 |

1. Wongpakaran N, Wongpakaran T, Wedding D, Gwet KL. A comparison of Cohen’s Kappa and Gwet’s AC1 when calculating inter-rater reliability coefficients: a study conducted with personality disorder samples. BMC Med Res Methodol 2013 Apr 29;13:61. PMID:23627889

2. Landis JR, Koch GG. The measurement of observer agreement for categorical data. Biometrics [Wiley, International Biometric Society]; 1977;33(1):159–174. [doi: 10.2307/2529310]
