# Supplementary material for: Platform Effects on Public Health Communication: A Comparative and National Study of Message Design and Audience Engagement Across Twitter and Facebook
Source: JMIR Infodemiology. 2022 Dec 20;2(2):e40198. doi: 10.2196/40198 (PMC9773105; doi:10.2196/40198)
Supplement: Multimedia Appendix 2 [file infodemiology_v2i2e40198_app2.docx]

**Appendix II**

Main annotation codebook and guidelines for coding.

|  | **Feature** | **Definition** | | **Examples** | | **NOT to Include** |
| --- | --- | --- | --- | --- | --- | --- |
| **Speech Function** | **Representative** | A sentence that is in declarative form describing a behavior, state or event related to the threat. This is the default category of a speech function. This category is not marked if a sentence is a directive or question prompt, however it may overlap with expressive or participatory request. | "#COVID19 can be spread by people who do not have symptoms & do not know that they are infected" | | *"Let's work together", NOR “Do you have symptoms of…?” NOR “Wear a mask when indoors”* | |
|  | **Directive** | A sentence that directs, commands or mandates an action. Generally in imperative form, such as "Donate to", "Wash your hands", "Wear a mask", "Get the facts", etc. Also mark in this category if there is a strong prescriptive statement: person "should" or "must" do something | "Help reduce the risk of exposure by..." OR "Call this number between 7:00 AM and 7:00 PM" OR "Continue to wear masks" OR "Donate blood." | | *Weak forms of directives, such as "you may call this number" or brief generic pointers to information such as, "Call:", "go to:", "learn more here" are NOT marked in this category: "Call:", "go to:", "learn more here"* | |
|  | **Question Prompt** | A sentence that asks a question but does not seem to expect an actual answer. If a question seems to actually ask a person to comment or participate in some activity *mark "request (for input or participation)" instead.* | "Working at a polling station?" OR "Are you looking for work? We are hiring!" | | *“What do you think about our recent policies - Could you add your comments below?* | |
|  | **Expressive** | Expresses a sentiment from the speaker, such as appreciation and honor, including symbolic phrases such as "celebrate the heros", "they are champions" or similar metaphors. It is usually in declarative form, but may be in directive (e.g. "be a hero"). Includes words "thank you" and "please". | "Thank you, #EMS heroes, for staying strong and fighting to preserve life" OR "It is a beautiful afternoon" OR "Good morning" OR "Be a hero" | | *"That is a great policy"; "It is better to use non-disposable…"* | |
|  | **Request (input/ participation)** | References to requesting people to participate in research, volunteer, or requesting people for opinions or input into an issue or policy (e.g. send your comments, Let us know what you think, etc.). | "Learn more information, including how to enroll [in the study]" OR "You are encouraged to donate blood here" | | *Does NOT include hiring requests NOR training offered by gov't. E.g. "Would you like to work for us?" NOR "We encourage you to seek help for your problems"* | |
| **Topic/Domain** | **Science (Threat cause, risk, mechanism or symptoms** | Describes or explains a cause, mechanism or impact/symptom of the main or related disease. Refers to scientific information, including medical or sociological description of facts and/or theories. Directions for how to protect or avoid threat is marked instead under *How-to-protect.* | "Currently, @FDAfood says there is no evidence that produce can transmit #COVID19." OR "Coronaviruses are named for the crown-like spikes on the surface of the viruses" | | *Does NOT include vague qualifications like "this disease is serious". NOR E.g. "We have information about covid-19 at this site..."* | |
|  | **Surveillance (Prevalence/ Statistics)** | Indicates statistics and data about prevalence (e.g. cases/deaths) of disease or related issue (e.g. statistics of lack of blood related to Covid-19, etc...). Statistics or rates of vaccines uptakes are also included here. Surveillance information about threat. | "Cases are rising in this county” OR “Yesterday, there were 85 new deaths”. | | *"Testing sites are available in this location tomorrow"* | |
|  | **Emergency event or recent policy change** | Sentence or message identifies an emergency event (of immediate concern, strong emphasis) or immediate need, or immediate policy change, or immediate closure. If the topic is a policy | "Travelers: DON'T book air travel to NY for just a few days or a week if you're from a state on the quarantine list. You will have to quarantine in NY for 14 days." OR "We need blood today" | | *"We have recently implemented a new app"* | |
|  | **Policy (actions, policies/programs)** | Actions, policies or programs of officials, government agencies or related entities. Includes references to "county govt" or "state govt" ("Our county is taking action to expand tests"). Includes reference to creating new webpages or new information, or reference to re-opening/closing policies, vaccine policies, provision of testing sites, etc. | "FDA is working around the clock to help expedite the availability of tests." OR "Multnomah County is almost ready for reopening schools." OR "We have created a new webpage here:" | | *Does NOT include generic qualification without specific kind of action (e.g. "We are making progress.") NOR a blank reference to "See update below". "We're here to help", NOR "We are making progress on being ready."* | |
|  | **Protection (How to protect or treat, efficacy)** | Information of what people can or should do to protect themselves from the main or related threat. It may be in directive or declarative form, or included in a question (e.g "Did you know you can do x to prevent y?"). Explain or recommends how to protect or treat COVID-19 or related issue. | "The best way to stop the spread of the #Coronavirus is to stay at home." OR "Clean and disinfect things you and your family touch frequently" OR “Wear mask” | | *Does NOT include policy action about testing/vaccines (e.g. "we have acquired vaccines" -- this is policy action). Also not including “testing sites are available here” etc.* | |
| **Type of Resource** | **Interactive resources** | Interactive information or communication service excluding an agency webpage. Examples include: phone numbers, hotlines, webinars, alerts, contact tracing, press briefing, apps, dashboards, etc. Message includes specific time and/or date, or reference to "watch live" or "was live". If live event was in the past, still mark category. | "Please reach out for help [phone number included in picture]" OR "Download the FREE COVID Alert PA app". "On Friday, May 15 from 1-2 p.m. ET, FDA will host a virtual Town Hall on the production &amp; use of 3D printed swabs" | | *"For more info see here:"* | |
|  | **Material** | Testing sites, vaccine locations, or programs that citizens can use (not merely online/phone). Should be relatively tangible and immediate resource available, such as financial or medical assistance. *This assumes interactive (no need to mark interactive as well if in this category).* | "The @CityOfBoston has expanded access to testing in several neighborhoods across the City. Use our map to find locations" | | *“We have a new dashboard”* | |
|  | **Corrective** | A correction of a rumor, or false information, or pointing to resources that address incorrect information. Rumor or myth correction should be explicitly referred to ("That is a rumor… that is a myth… etc."), or its references directedly linked to. | "For help in distinguishing rumor from fact, see @FEMA Coronavirus Rumor Control: " OR "NOTE: A death previously reported in Warren was incorrect, and has been removed." | | *"There is misinformation out there, be careful!" NOR https://www.facebook.com/VDHgov/posts/1530434970460845* | |
| **Audience** | **Population group** | Refers specifically to a demographic group (e.g. adults, hispanics, etc.), or a vulnerable population (e.g. elderly, patients with cancer, low-income groups, etc.). Includes messages directed toward a particular kind of profession, or business, such as labs, research centers, health depts, etc.) | "Cancer patients are among those at high risk of serious illness from a COVID19 infection." OR "Are you homebound because of COVID-19?" OR "Researchers, Clinical Labs & Commercial Manufacturers:" | | *Does not include "location based" publics (e.g. Ohians, etc.), and does NOT include if post is simply on another language. "Ohians must act together" NOR "If you are feeling sick or anxious"* | |
| **Threat Focus** | **Secondary Threat** | Consequences or issues directly related to the threat, such as lack of tests, vaccine shortage, "infodemic", domestic violence, mental health, etc. If message mainly about a related issue (e.g. food bank) in the context of Covid-19 also mark here. | "Many are feeling stressed because of #COVID19." OR "Emergency department indicate some patients may be delaying emergency care during the #COVID19 pandemic" , etc. | | *Does not include messages about symptoms of the disease. "The following are symptoms of COVID-19: …. "* | |
| **Speaker/Agent** | **Agency expert/staff** | Agency expert (person), or staff member is quoted on, is the agent or main speaker mentioned in the message, such as a relevant doctor or main head of agency. | “The first step... is to get to know all of the partners involved,” says CDC’s Maggie Silver. " | | *“U.S. Surgeon General Dr. Jerome Adams shows how to make…” when Posted by Delaware State Health Dept.* | |
|  | **Political actor** | Political actor, such as a governor or mayor, is described as agent of message, doing something, is quoted or speaks about the issue *Does NOT include instance where @mayor tag, etc. is simply included at end of message.* | "Watch the Mayor’s updates on https://t.co/TUqXs1b5Bk " | | *"Covid is rising by 30%. This is our update. #Covid-19 @MayorDungan ”* | |
|  | **External agency/expert** | Agent or speaker is another agency, organization or an external expert or individual providing service or speaking about issue (e.g. "CDC says that", or "Walgreen is offering free testing") | "... sits down with the head of @samhsagov Dr. Elinore McCance-Katz to discuss the crisis" | | *When message speaker actually works for the agency.* | |
|  | **Personality /Celebrity** | Social figure such as a film or sports celebrity speaks the message, is mentioned as speaking, or is the main agent of the action/policy mentioned. Includes personal covid story sharing or individual perspective, such Army Lieutenant or little baby girl. | "Juan from Blue Eagles football club speaks about COVID19" OR "First Lieutenant Jacob Sommerfeld shares his covid story" | | *Someone like Tony Fauci speaking. He is not considered a celebrity here.* | |
| **Rhetorical Tactic** | **Collective frame** | Message increase collective efficacy or implicates the whole community or in the message. References to "we can do this together", "we are united", "everyone needs to help", etc. Includes reference to helping "community", "neighbors" and "family". | "We all need to do our part to combat Covid-19”. “We can all help our community” | | *"We're here to help" NOR "We need you to donate blood today"* | |
|  | **Positive agency action** | Action of agency is framed in a clearly positive way. Includes explicit positive adjective or verbs such as "working hard" or "we are here to help", etc. Clearly highlighting positive actions of its own staff. *More objective phrases such as "We have tests available", is NOT considered here.* | "FDA is working around the clock to help expedite the availability of tests." OR "We're here to help" OR “We’re making progress is getting vaccines” | | *"We have tests available"* | |
|  | **Strong emphasis** | Emphatic or exclamatory sentence, usually to emphasize danger or great risk, or outcomes. Use of strong adjectives such as "it is critical that we", "vital", "necessary", "dramatically", "enormous", "we need you", "the most dangerous", etc. Also includes use of exclamation points and all caps in text. | "#COVID19 cases are increasing dramatically." OR " OR "Answer the call! Public health contact tracers ..." | | *Terms like "highest" or "lowest"(terms of precision) are NOT considered here if they are factual (e.g. we have the highest rate in the country). E.g."We have the highest case rates yet during the pandemic"* | |
|  | **Metaphor** | Sentence or message uses metaphors to explain the disease mechanism, threat or risk, such as thinking of "swiss cheese" as layers of protection or other non-technical description of threat or risk. | "The swiss cheese respiratory virus defense" | | *"This disease is dangerous"* | |
| **Media** | **Text-in-image** | The post has an image where the image adds new or additional information not expressed on the main text of the message. This includes a link that is on the image, but not on the text. Consider this category for image shown on post that may be from an external link. | [Any text in the image that adds information or message not included in the original text of the message] | | *Does NOT include logos or brands (e.g. Nebraska: Good life, good mission).* | |
|  | **Illustration** | Any use of image with any amount of illustration or design, even if only background or small use of icon. If an image comes from a link and is displayed as part of the post, you may mark here. *Does not include logo.* | [https://twitter.com/PAHealthDept/status/1252257837432135681;](https://www.facebook.com/HealthVermont/posts/3863771653657603) | | *Does NOT include instance of an image with solid color background and simply text on it. Does NOT include video caption/frozen image.* | |
|  | **Photo-graph** | Any actual photograph of object or person. If photograph has some illustration, you may mark photograph AND illustration. | [https://twitter.com/StateHealthIN/status/1277240842869182464;](https://www.facebook.com/DouglasCountyHealth/posts/2751274534985488) | | [*https://twitter.com/HealthyFla/status/1260953060400119815*](https://twitter.com/HealthyFla/status/1260953060400119815) | |
|  | **Infographic** | Any image with a graphic that conveys data (e.g. line graph, map). Can also be at least one statistic (e.g. total, count, %, etc.) and one piece of illustration (including an icon); OR at least 2 descriptive (instructional) graphics with text. | [https://twitter.com/DHSWI/status/1311742770177019905;](https://www.facebook.com/FargoCassPublicHealth/posts/10156745458162624) | | [*https://twitter.com/KCHealth1/status/1341005498737254400*](https://twitter.com/KCHealth1/status/1341005498737254400) | |
|  | **Video** | The post has a video included in the message. | [Any video that appears OR is an embedded as part of post] | |  | |
| **Language** | **Other Language** | Message contains at least one sentence in language other than English. Identify language: Spanish, Arabic, etc. or X if unsure. | “Números del #COVID19 en California:” | |  | |
| **Basic Rules for Coding** | | | | | | |
| 1 | Analysis is done together on each sentence and the overall message. Text on image (except brief slogans or logos) are considered for coding. | | | |  | |
| 2 | Analysis should be done on original tweet/post via the status URL. | | | |  | |
| 3 | Every category is binary: 1 or 0 for whole post. | |  | |  | |
|  | Except: Language should have language name or 1 if not English. | |  | |  | |
| 4 | Generally, within every dimension, the specific feature (also called element or category) is mutually exclusive for a single sentence or clause. However, this is naturally not always the case. See recommendations within definitions and examples of features. | | | | | |
| 5 | Hashtag meaning are NOT considered as part of content analysis, except: when they clearly complete a sentence. | | | | | |
| 6 | Coding for all features are for relatively explicit content and not inferable or implicit. For example, a message that simply states “*update on Covid-19 here”* is not assumed to be about *disease mechanisms,* and potentially not any topic (although a primary focus on Covid-19). | | | | | |

Illustrative examples of messages with annotated features.

Exhibit 1. Wisconsin Department of Health Services

Expressive (speech function)


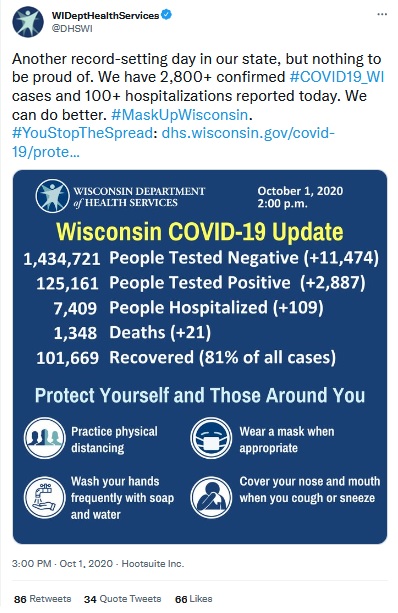


Hyperlink (informational resource)

Representative (speech function)

Prevalence/Statistics (topic)

Collective frame (rhetorical tactic)

Directives (speech function)

How-to-protect (topic)

Text-in-image (media)

Infographic (media)

Exhibit 2. Pennsylvania Department of Health

Representative (speech function)

Action/Policy (topic)

How-to-protect (topic)

Material Resource (resource type)


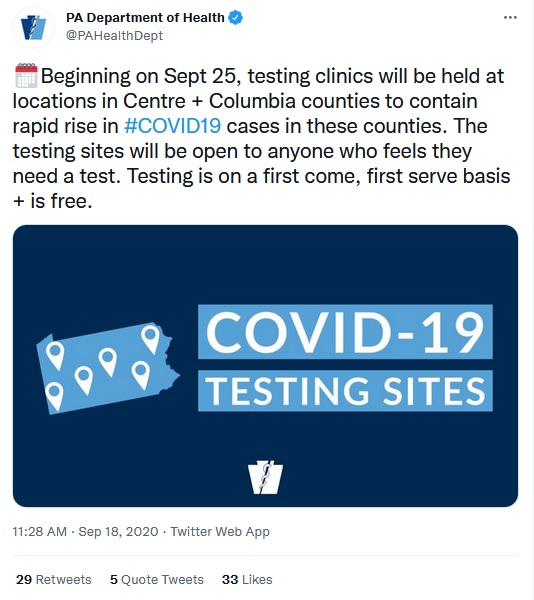


Illustration (media)*

*Text-in-media is *not* considered here, since there is no additional information from main text.

Prevalence (topic)

Exhibit 3. North Carolina Department of Health and Human Services

Representative (speech function)

External agency (speaker)


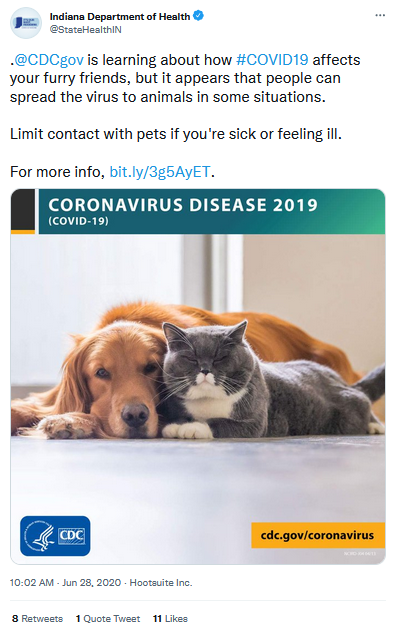


Hyperlink (media/informational resource)

Directive (speech function)

How-to-protect (topic)

Photograph (media)

Scientific (topic)
