# Supplementary material for: Platform Effects on Public Health Communication: A Comparative and National Study of Message Design and Audience Engagement Across Twitter and Facebook
Source: JMIR Infodemiology. 2022 Dec 20;2(2):e40198. doi: 10.2196/40198 (PMC9773105; doi:10.2196/40198)
Supplement: Multimedia Appendix 3 [file infodemiology_v2i2e40198_app3.docx]

**Appendix III**

Below are the statistical formulas used to assess normalized likes and shares.

The normalized likes (*NL_m_*) measure of a message *m* is:

$${NL}_{m}=\frac{L_{m}}{F_{a}} \times100$$

where $L_{m}$ is the number of likes of the particular message, and $F_{a}$ is the follower count of the account that posted the message. NL*_m_* is the percentage of the agency’s follower count that liked the message.

A similar measure was created for normalized shares (NS) of a message *m*:

$${NS}_{m}=\frac{S_{m}}{F_{a}} \times100$$

where $S_{m}$ is the number of shares (retweets) of the particular message, and $F_{a}$ is the follower count of the account that posted the message.

Below are detailed results from the mean and interquartile ranges of followers per account, as shown in Figure 1 of the manuscript.

|  |  | Local | State | Federal | All |
| --- | --- | --- | --- | --- | --- |
| **Facebook** |  |  |  |  |  |
|  | Mean | 14,651 | 74,464 | 602,601 | 106,365 |
|  | Min | 921 | 2,185 | 16,430 | 921 |
|  | First | 3,913 | 22,488 | 148,516 | 8,685 |
|  | Median | 6,971 | 61,529 | 279,248 | 31,573 |
|  | Third | 23,068 | 89,702 | 457,050 | 83,481 |
|  | Max | 92,760 | 285,224 | 3,294,226 | 3,294,226 |
| **Twitter** |  |  |  |  |  |
|  | Mean | 14,754 | 26,234 | 959,582 | 123,430 |
|  | Min | 811 | 1,221 | 31,914 | 811 |
|  | First | 3,522 | 9,921 | 69,224 | 6,946 |
|  | Median | 6,941 | 19,312 | 415,560 | 17,362 |
|  | Third | 14,407 | 34,946 | 1,313,749 | 35,313 |
|  | Max | 86,587 | 90,450 | 3,355,778 | 3,355,778 |
